# Alarming Hemorrhage after Tonsilar Excision

**Published:** 1887-05

**Authors:** 


### *ALARMING HEMORRHAGE AFTER TONSILAR EXCISION.*

Hemorrhage after the excision of the tonsils is so rare that the following case, reported by Dr. Clinton Wagner in the *New York Medical Journal* for April 16, 1887, deserves attention. In a previous paper, Dr. Wagner had stated that he had performed the operation 500 times without accident. Hemorrhage, however, does sometimes occur, and it is necessary to be on the lookout for it, as this case shows. The report is as follows :

"Madame B. F., aged about 30, an opera singer by profession, consulted me for sore tonsils. I found the left tonsil greatly enlarged, and she informed me that it frequently became actually inflamed. I recommended excision. The gland was partly covered anteriorly by the column of the soft palate, and extended so far downward into the pharynx that I had great difficulty in encircling its inferior portion within the ring of the guillotine. Finally, I succeeded in removing almost the entire gland. A gush of blood followed, but apparently not greater in quantity than is usual after this operation. The bleeding increasing instead of lessening, I applied to the cut surface of the gland the persulphate of iron, which, failing, I resorted to compression, but I discovered afterward that it had not beene xerted in the proper direction. Nearly an hour had elapsed since the operation, a large amount of blood had been lost, vomiting of blood, which had found its way into the stomach, ensued, and the patient was rapidly losing strength. I cleansed the parts thoroughly of clots of blood, but not finding the source of bleeding, I forced the tongue, by means of the depressor, upon the floor of the mouth as far as possible. In the space between the pillar of the soft palate, apparently springing from the root of the tongue, I discovered an artery, of considerable size, bleeding freely, and with such force that the blood was projected over and beyond the depressed tongue to the opposite side of the mouth. The bleeding vessel, now located, was, without much difficulty, taken up with an artery-forceps and twisted, and all further hemorrhage was effectually controlled. I think the divided artery was either the tonsilar branch of the facial, or the largest of the branches of the ascending pharyngeal, both of which are given off from the external carotid."

